# Evaluating student nurses satisfaction with educational escape rooms as a pedagogical approach to teaching professional nursing values

**DOI:** 10.1186/s12912-025-03912-1

**Published:** 2025-10-21

**Authors:** Nicola Rowley, Sandra Lucas

**Affiliations:** School of Health and Life Sciences, University of the West of Scotland, Scotland, UK

**Keywords:** Nursing students, Escape rooms, Nurse education

## Abstract

**Background:**

Understanding the expectations of new generations of students entering higher education can be challenging. Generation Z have been brought up in a digital world, keeping them focused and entertained. Education must respond to the changing student profiles. Consideration should be given to integrating a hyper-cognitive approach to meet the expectations of students and fuel a positive learning experience for all evolving generations.

**Aim:**

To determine student nurses’ satisfaction of escape rooms to facilitate learning of professional values; teamwork, decision making and sharing information.

**Method:**

Quantitative cross-sectional questionnaire to capture participants’ satisfaction following their participation in the escape room activity.

**Framework:**

The pedagogical benefits of escape rooms were explored as a teaching strategy for the evolving generation of students entering nurse education. The premise being,offering influential ‘games’ which nurture and encourage active collaboration among students would help to form peer connections and create relationships among new students entering nurse education. Escape rooms are coactive and can drive interdependence among participants sharing a goal by creating an environment for team working in nursing education.

**Results:**

Of the 45 student nurses 100% (n=45) felt part of a team, 98% (n=44) had a positive experience, and only 2% (n=1) thought that the experience was stressful. Conclusion: Using escape rooms as a teaching and learning tool is an innovative pedagogical approach, offering a dynamic, hands-on experience that engages students through problem-solving and teamwork. Student nurses gave positive responses to teamwork, decision making, sharing information and enjoyment of the activity. This research will inform the development and uptake of core competencies for future nurses, creating teamwork and professionalism within a collaborative live educational environment.

**Clinical trial number:**

Not applicable

**Trial registration:**

Pilot not a controlled trial. No registration.

## Background

Effectively engaging Generation Z students has become a growing focus for university lecturers [1, 2]. Students accustomed to a world of pervasive technology and instant access to information often present challenges in areas such as focus and work ethic [1, 3]. Generation Z, born between 1997 and 2010, have been raised in a digital age fuelling different learning styles and expectations compared to previous generations [4, 5]. There is a need for educators to adapt teaching methods to effectively engage this new wave of students [6–8], and provide appealing learning environments which motivate and inspire students. Nursing educators can align teaching methods by incorporating simulation labs and experiential learning opportunities that allow students to apply theoretical knowledge in real-world settings [6] and develop essential professional skills for nursing [9].

Delivery of nurse education continuously evolves to meet the demands of nursing professionalism and accountability from governing bodies. Within higher education teaching and learning in nursing has advanced with new technologies, virtual reality, and simulation [10, 11]. Whilst these strategies have well-defined advantages, all of them can, in theory, be undertaken independently. Generation Z come with skills of independence, often fuelled by social media and ready access to a digital world. However, this self- governing can cause undeveloped social and relationship skills [12, 13] both of which are considered essential attributes within the nursing profession [14, 15]. Nursing programmes recruit students across a wide age range and although the focus of this study was placed on the challenges of younger students to engage in traditional lecture style learning, escape room activities can be suitable for all generations [16]. There was no intention to exclude Gen X or Gen Y from progressive teaching methods but instead to explore a strategy to better engage Gen Z to work alongside generations of nursing students. There have been several studies which have advocated that a teaching and learning environment which drives co-dependence can also develop essential skills such as teamwork, communication, and problem- solving shared goals [17, 18] thus preparing students for the professional standards of nursing.

The interactive experience of escape rooms promotes co-dependence among participants as they work toward a common goal. This environment can be an effective tool for team-based problem-solving within nursing programs [19]. There is some indication in the literature that educators have adopted the problem-solving format of escape rooms to engage students, foster critical thinking, and support collaborative teaching methods [20] It has also been reported that escape room games align with constructivist theory which can enhance nursing students’ ability to apply theory in clinical settings, promoting safer learning, and improving patient safety [21]. By investigating the effectiveness of this innovative method of teaching, educators can better prepare nursing students for the complex, collaborative, and fast-paced nature of healthcare, ultimately leading to improved patient care and safety [18].

## Methods

### Research aims

To determine student nurses’ satisfaction of escape rooms to facilitate learning of professional values; teamwork, decision making and sharing information.

### Sample of participants

The cohort of students selected for the escape room activity were articulating from an HNC programme. The HNC programme typically offers a gateway to Higher Education among younger people as an alternative gateway of the SWAP (Scottish Widening Access Programme) is not available to school leavers. The students articulated into Higher Education for four different Further Education Colleges although all had completed the same HNC programme within the year of articulation. The age range of the students was not predetermination, however based on the nursing pathways available within Further Education it was likely that the articulating students would belong to a younger generation. The number of students who achieve articulation into year 2 of Higher Education varies yearly. Therefore, a review of the number of articulating students over the last 5 year was taken, which showed an average of 39.2 students per year. This number was considered acceptable to complete this pilot study. In 2023 the number of students who had received a conditional offer for direct entry to year 2 of both the pre-registration Adult Nursing and the Mental Health Nursing BSc (Hons) Nursing programme was 45 in total. The premise for attending a Higher Education Institution was to develop knowledge, skills and professional values within healthcare environments that would enable students to demonstrate that they have met the requirements for progression into year 2 of the Adult or Mental Health Nursing Programme.

### Methodology

A quantitative cross-sectional questionnaire was utilized to measure participant satisfaction at a single point in time. This approach was cost effective and allowed for robust statistical analysis. Although this method only captures a snapshot of one event at a single point in time it minimized variability and reduced the risk of cohort differences affecting demographics.

The questionnaire used in this study was a modified version of the Readiness for Interprofessional Learning Scale (RIPLS) [22]. RIPLS comprises of four dimensions that explore attitudes towards teamwork, collaboration, and shared learning, elements that closely align with the aims of this study. Although originally designed for interprofessional learning, RIPLS places emphasis on professional values and interdependence, making it a relevant tool in this context. Created for undergraduate healthcare education, the RIPLS questionnaire evaluates students’ readiness for interactive, shared learning and has been validated by subsequent studies as an effective tool for assessing student attitudes toward multiprofessional education [23, 24].

To improve the validity, reliability, and clarity of the modified questionnaire, it was piloted with five academics involved in nursing education at the intended level. Feedback on the structure, relevance, and clarity was sought for all 20 questions, and no changes were recommended.

Participants, all enrolled in the BSc Adult and Mental Health Nursing Programme, engaged in an escape room activity during their first day at the University in May 2023. During the pre-briefing, students were informed that paper questionnaires were available to complete anonymously in an allocated room and could be left on a designated table. Paper questionnaires were used as it was not possible to access online questionnaires within the simulation rooms.

A single round of data collection was conducted, capturing a snapshot of students’ experiences of teamwork, decision-making, and information sharing during the activity.

The questionnaire focused on participants’ satisfaction with the escape room activity and consisted of 20 questions, divided into four themes: teamwork, decision-making, information sharing, and overall experience.

Responses were collected using a reliable Likert scale [23], which enabled structured data for efficient analysis, despite the known risk of response bias, such as central tendency or avoidance of extreme options [25, 26]. Closed questions were used to reduce participant anxiety about providing ‘correct’ answers [27], while a final open-ended question allowed for some qualitative feedback [28].

### Data analysis

Results of responses to each question were inputted into Excel and descriptive statistics using central tendency summarized the distribution of responses to identify the most frequent response [29]. The data was analysed using mean for an overall trend and mode for identifying the most popular or frequent response. Together, they provide a fuller picture of the data, allowing for an understanding in both the central tendency and the distribution of responses.

## Framework

### Escape room activity

Escape rooms were used as a teaching strategy to build confidence in collaborative working [30] and facilitate peer connections prior to participating in clinical simulation [31]. It was hoped that being immersed in the backdrop of escape rooms, participants would be able to effectively collaborate in a team environment, apply decision-making techniques to solve complex problems, and contribute to the transparent sharing and management of information to enhance team performance and decision outcomes.

**Fig. 1:**
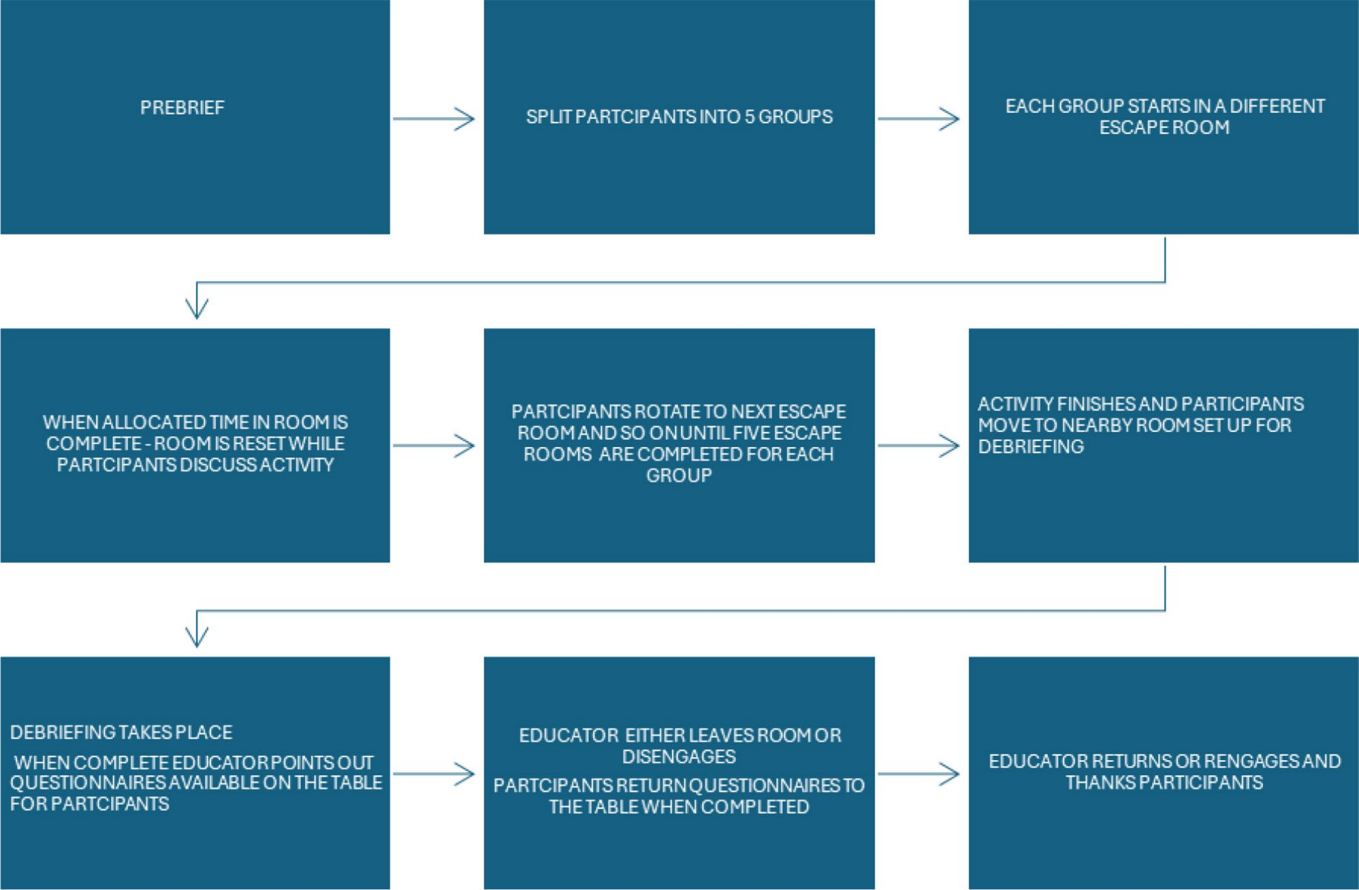
Phases of activity

The phases of activity (Fig. 1) were conducted identically and concurrently across two campuses of the University of West of Scotland, with trained facilitators at each location. The escape room facilitators were trained by the researcher to ensure consistency in delivery and identical trainer guidance was positioned within each escape room across each campus.

### Pre information

Students received Participant Information Forms (PIS) outlining the escape room activity and the research aims to ensure informed consent of participation. The PIS emphasized that students could opt out of the activity and/or the research without affecting their learning experience. Consent forms were completed before the activity, and non-participating students had access to alternative study materials. Completion of post-activity paper questionnaires were entirely voluntary, with non-completion having no impact on the participants experience. To highlight the voluntary aspect of the escape room activity students were encouraged to ask open questions and discuss their understanding of the PIS. This approach can help to make the decision making of potential participants more personal and eliminate coercion [32].

### Pre-briefing

A pre-briefing session addressed potential misconceptions about escape rooms and ensured psychological safety. Whilst no specific tool was adopted the pre brief was centred around the context of the activity. Open questioning helped students understand their roles, discuss their expectations and develop shared learning outcomes. Confounding factors, including prior experiences and stress levels, were also addressed during the pre-brief. Of the 45 students, only three had prior escape room experience which was in a recreational setting.

### Escape room design

**Fig. 2:**
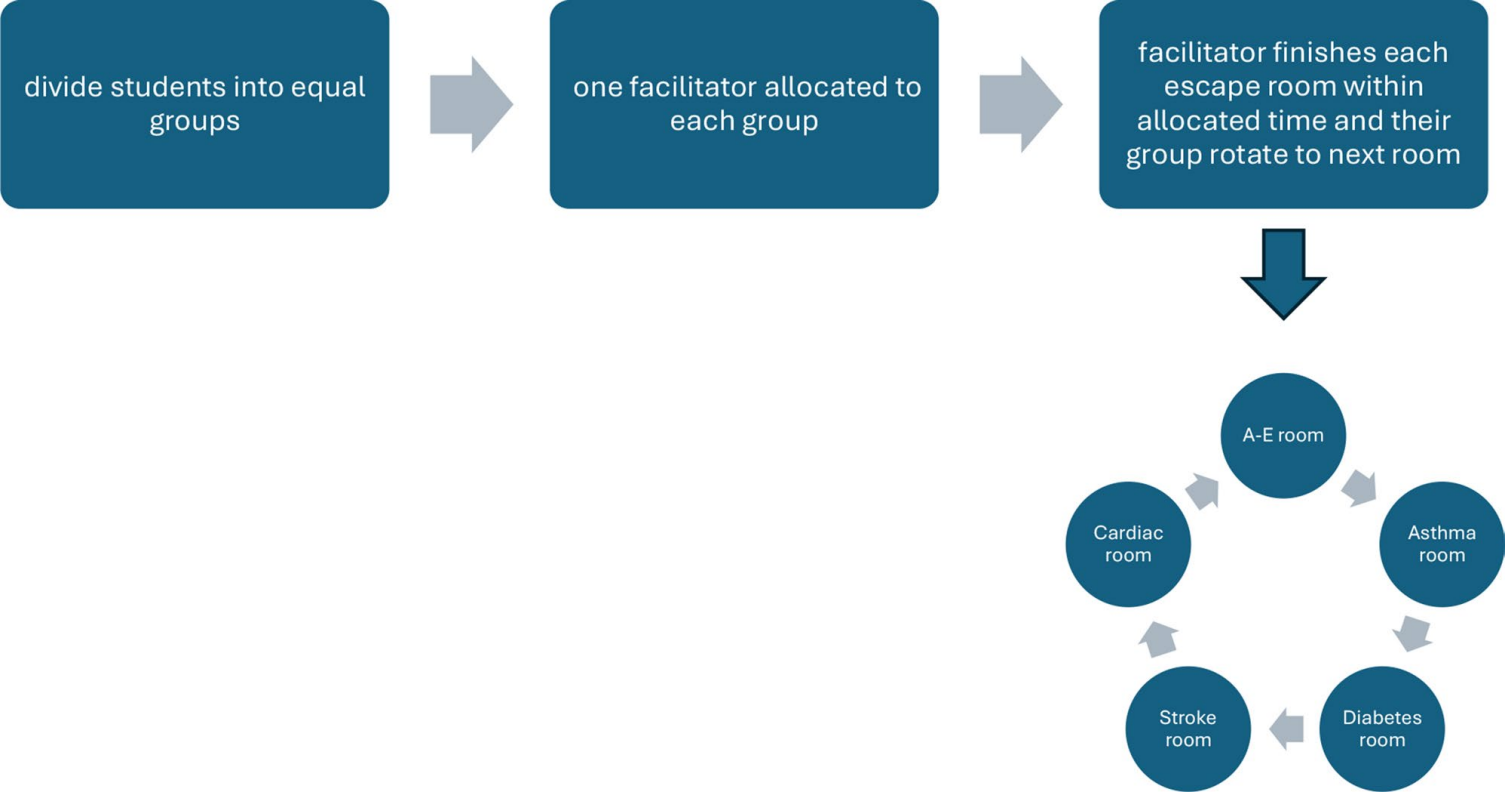
Stages for delivery of escape rooms

Five escape rooms, designed within skills labs, focused on diseases and conditions such as asthma, diabetes, stroke, cardiac arrest, and A-E patient assessment (Fig. 2*Stages for Delivery of Escape Rooms*). To minimize costs, basic resources like whiteboards, labels, and medical equipment were used. Each escape room featured written scenarios with problem-solving tasks that required identifying clinical signs, selecting treatments, and deciding on appropriate equipment. Facilitators provided guidance and ensured tasks were completed accurately, using standardized answer sheets.

To alleviate potential anxiety, students were not locked in the rooms but given timed challenges to maintain engagement and encourage quick decision-making. Groups of 5–6 students worked collaboratively under the supervision of one facilitator per room.

### Debrief

After the activity, a debriefing session allowed participants and the facilitator to reflect on the experience and share feedback. The method of ‘Debriefing with Good Judgement’ was utilised to encourage participants to explore their driving frames, knowledge and assumptions of their experience [22] Using a ‘Triumphs and Challenges’ activity on a whiteboard, students were encouraged to share their immediate thoughts. A facilitator summarised the challenges, discussed learning points, and highlighted ‘take home messages.’ Debriefing was key to fostering collaboration, reflection and consolidating a meaningful learning experience. The debriefing was combined facilitator and participant led therefore the discussion was unpredictable. Initiating discussion under the headings of ’triumphs and ‘challenges’ allowed for a more balanced reflection and was less coercive in unravelling specific opinions of the experience.

### Questionnaires

The ‘triumphs’ and ‘challenges’ during the debriefing did not highlight specific topics however the points were summarised into ‘take home messages’ which were agreed amongst the participants. These ‘messages’ may have influenced participants choices on the questionnaire however participants experiences are unpredictable therefore this is unavoidable. Conversely, debriefing is a group discussion, with personalities of participants sometimes impacting on levels of participation therefore the anonymity associated with the questionnaire may have facilitated more open and honest answers.

## Results

### Demographics

All student participants had attended a Further Education (FE) college within Scotland and achieved A/B within the graded unit of the HNC in Healthcare Practice programme. This enabled articulation to year 2 of the UWS BSc Adult Nursing Programme. The 45 students articulated onto the BSc Adult Nursing Programme, and 45 students participated in the escape room activity on their first day of attendance in 2023. 43 students were female and 2 were male. Generation Z accounted for 27 students, 17 students were millennials, and 1 student was generation X. It is interesting to note that the mean age of student nurse participants was 29.1 years, 2 years outside the eldest age of generation Z. Whilst most participants (57.8%) were from generation Z, 37.8% of students were millennials and 4.4% generation X.

The geographical area in which students had attended FE colleges was widespread across Scotland; Lanarkshire (17 students) Ayrshire (9 students), Renfrewshire (8 students) and Dumfries (2 students). A summary of participant demographics is presented in Table 1 Demographic Data.

**Table 1:** Demographic data

	Gender		Means Age	Generation			Location				
	Female	Male		Gen Z	Millennials	Gen X	LK	Ayr	Ren	Dum	other
Number of participant (n=)	43	2	29 years old	27	17	1	17	9	8	2	9
Percentage (%)	95	5		58	38	4	38	20	18	4	20

**Key** LK = Lanarkshire, Ayr = Ayrshire, Ren = Renfrewshire, Dum = Dumfries, Other = not disclosed

### Questionnaires

The results from questionnaires (Table 2 questionnaire results) stated there was a positive impact on team building. The highest response rates were related to the question of feeling part of a team with 100% of students indicating that they agreed. Sharing learning opportunities and feeling listened to during the activity also yielded positive results of 98% students agree respectively.

Student nurses’ responses to an improvement in effectiveness when working together showed that a 100% of students agreed that their peers enabled their own effectiveness throughout the activity. While encouraging professional values such as trust and respect between students yielded 98% of students in agreement and 91% of students’ nurses agreed that the activities with the escape rooms were appropriate to their stage of learning.

For problem solving the student nurses experience of the escape rooms yielding positive responses with 89% of students enjoying the activities.

Involving fun and difficulty 94% of students indicated that the escape rooms were fun. All student nurses (100%) agreed that the escape rooms were not too difficult with only 2% indicating that they experience a degree of stress.

The additional comments section at the end of the questionnaire provided an opportunity to understand the lived experience of the student nurses.

great team building’ ‘fab start to the EPLE module’ ‘really enjoyed this experience.

During the debriefing it was apparent that new relationships and mutual trust had been formed amongst student nurses who were not known to each other three hours prior to the escape room activity. The use of the escape room activity encouraged and formed connections to each other resulting in 93% of participants indicating that they would welcome escape rooms within other modules. Moving forward it may be beneficial to review the activities integrated within the escape rooms to ensure the knowledge and understanding required is appropriate to the current level of learning.

**Table 2:** Questionnaire results

	Questions	Corresponding themes	Strongly agree/agree *n* = (%)	strongly disagree/disagree *n* = (%)
1	Working with other student nurses helped me to be a more effective within an escape room.	Teamwork, decision making and sharing information	45 (100%)	0
2	Student nurses need to trust and respect each other to work within escape rooms.	Teamwork and decision making	44 (98%)	0
3	Sharing learning helped me understand my own limitations within the escape rooms.	Teamwork and sharing information	44 (98%)	0
4	I felt listened to within the escape room activity.	Teamwork, decision making and sharing information	44 (98%)	0
5	I felt part of a team within the escape room activity.	Teamwork	45 (100%)	0
6	I liked the mix of activities within the escape rooms.	Experience	42 (93%)	0
7	The activities in the escape rooms were appropriate to my stage of learning.	Experience	41 (91%)	0
8	I enjoyed the problem-solving activities within the escape rooms.	Experience	40 (89%)	0
9	The supervision of the escape rooms was adequate.	Experience	44 (98%)	0
10	I was given enough information to understand the objective of the escape rooms.	Experience	44 (98%)	0
11	The time within each escape room was adequate.	Experience	44 (98%)	1 (2%)
12	I wish I had received more help within the escape rooms.	Experience	0	44 (98%)
13	The escape rooms were stressful.	Experience	1 (2%)	42 (93%)
14	The escape rooms were too hard.	Experience	0	45 (100%)
15	The escape rooms helped with team building.	Team building	44 (98%)	0
16	The escape rooms were fun for me.	Experience	42 (94%)	0
17	I would welcome escape room activities within other modules.	Experience	42 (93%)	0

## Discussion

This discussion explores the findings of the study, focusing on participant satisfaction of escape rooms to foster professional values such as teamwork, decision-making, and information sharing among student nurses.

### Themes

The analysis of this pilot study was guided by corresponding themes: teamwork, decision making, sharing information and the overall experience of the activity. The themes were built from related questions on the post activity questionnaire and all questions were constructed to address the aim of this study and present a clear correlation with the data collection tool.

### Generational similarities and differences

The generational composition of the participants provides an intriguing lens through which to interpret these findings, highlighting the influence of diverse learning preferences across Gen Z, GenY (millennials), and Gen X. It has been suggested that Gens Y and Z share similar values, particularly in their need for leadership. However, differences and similarities in work values and communication have been identified among the three generations of clinical nurses [33]. Whilst it is important to recognize and address these differences across the generations, this pilot study highlights that values-based learning activities such as escape rooms can develop affective domain learning and support the socialisation of students into the nursing role [34]. The generational composition of the participants offers valuable context for understanding the broader applicability of escape rooms as a teaching strategy. With a mean age of 29.1 years, the cohort included a mix of Generation Z, Millennials, and Generation X students, each bringing unique perspectives and learning preferences shaped by their generational experiences. Generation Z students, who have grown up in a digital and interactive world, often prefer hands-on and collaborative learning approaches, making them well-suited to dynamic activities like escape rooms [12]. In contrast, Millennials and Generation X learners may place greater value on structured, team-oriented educational experiences. This generational diversity highlights the importance of designing flexible, inclusive teaching strategies that cater to varying preferences while fostering collaboration and professional skill development. Escape rooms, by their nature, provide an engaging platform capable of bridging these generational differences in learning styles, enhancing their utility in diverse educational settings. The purpose and goal of escape room should be carefully considered as activities need to align with the desired outcome for both participants and educators. Pedagogies that promote team-based or collaborative learning can be step based to achieve a goal, creating co-dependence between the players where as more linear activities may achieve delegation and leadership [40]. 

### Participant anxiety

Lack of reported stress was a surprising result, which challenges common assumptions on simulation-based learning being a source of anxiety and stress [41–43]. It was preempted that the unfamiliarity of the simulation environment the escape room activity could have evoked anxieties among participants therefore a detailed pre-brief and comprehensive debrief was put in place. This study participants reported no stress which underscores the significance of taking a structured and holistic approach to escape rooms as an innovative teaching strategy. Existing literature on safer learning environments suggest activities involving time constraints and problem-solving tasks can often induce anxiety, yet the structured and supportive environment of the escape rooms appeared to mitigate this potential challenge. Literature also suggests that well-facilitated experiential learning activities, like escape rooms, can balance the pressure of tasks with a sense of psychological safety, allowing participants to focus on collaboration and problem-solving rather than individual performance [30]. This result challenges assumptions that high-energy, time-sensitive learning scenarios inherently lead to stress, highlighting the importance of careful activity design and facilitation. The ability to engage students in a stimulating yet non-threatening manner may enhance the effectiveness of escape rooms as a pedagogical tool, especially for Generation Z learners, who value both challenge and support in their educational experiences [12]. This finding underscores the potential of escape rooms to provide a balance of excitement and engagement without compromising the well-being of participants.

### Professional nursing values

Teamwork is a cornerstone of nursing practice, and fostering this skill among student nurses is essential for their professional development. Escape rooms provide a unique opportunity to simulate real-world collaborative environments, requiring participants to rely on one another to achieve shared goals. Literature emphasizes that such activities cultivate teamwork by encouraging active communication, mutual trust, and collective problem-solving [17]. These collaborative experiences align with the interpersonal demands of nursing, where effective teamwork is directly linked to improved patient outcomes [18]. Furthermore, the interactive and engaging nature of escape rooms addresses the learning preferences of Generation Z students, who benefit from dynamic, team-oriented activities that mirror professional settings [12]. By integrating teamwork into a structured yet flexible format, escape rooms offer a practical approach to preparing nursing students for the collaborative challenges of clinical practice and developing as a professional.

The development of decision making is a critical aspect of nursing education, shaping students’ understanding of their roles, responsibilities, and values within the healthcare system. Escape rooms provide an innovative platform for fostering and developing decision making skills by engaging students in scenarios that emphasize core nursing principles such as accountability, ethical decision-making, and patient-centered care. Literature supports the use of interactive learning strategies, like escape rooms, to reinforce professional values by immersing students in realistic, high-pressure situations that require them to embody their professional roles [25]. Such activities promote reflection on their actions and decisions, helping students internalize the attitudes and behaviors expected of nurses [16]. Additionally, shared learning within the escape rooms encourages students to understand the benefits to expanding opportunities to learn from their peers as well as fostering mutual respect and a shared responsibility. This alignment of theoretical knowledge with practical experience supports the cultivation of a strong professional identity, preparing students to navigate the complexities of clinical practice confidently.

### Experience of escape rooms

The experience of participating in the escape room activity was characterized by high levels of engagement, enjoyment, and immersion, contributing to its effectiveness as a teaching strategy. Escape rooms offer a dynamic, hands-on approach to learning that contrasts with traditional methods, providing students with an opportunity to actively apply their knowledge in a collaborative and problem-solving environment. Research highlights that such experiential activities enhance motivation and retention of learning by creating memorable, real-world scenarios [35, 36]. Additionally, the structured yet flexible format of the activity allows students to engage at their own comfort level, fostering a supportive atmosphere that promotes both individual and group participation [37]. The novelty and interactivity of the experience align with the preferences of Generation Z learners, who thrive in environments that prioritize active involvement and immediate feedback [13]. Overall, the positive response to the escape room activity underscores its value as an engaging, straight forward and effective tool for facilitating learning and building professional skills for nursing. However, it is worth giving important consideration to the academic and social skills of the participants of escape rooms. Students who are unable to complete activities with the escape rooms can become frustrated and critical about their learning experience [38, 39]. 

## Limitations

This research had several limitations, including the sample size, questionnaire design, and recruitment process. While the small sample size could potentially impact the generalisability of the findings, similar studies exploring students’ attitudes toward escape rooms as a learning environment have utilized participant numbers ranging from 14 to 42 and still yielded credible and valuable insights [37, 44]. This suggests that, despite its size, the sample in this research aligns with established norms for research in this area. Additionally, efforts were made to counter the potential limitations of the questionnaire design. Clear and focused questions were formatted to directly address the aims of the research, minimizing the likelihood of misinterpretation and ensuring relevant data collection [45]. While the reliance on closed questions limited the depth of analysis, it allowed for a 100% completion rate and straightforward data analysis. The possibility of response bias, such as the tendency to avoid extreme categories, was acknowledged [26], but this is an inherent limitation of Likert-scale-based research. Despite these limitations, the findings provide valuable insights into the pedagogical potential of escape rooms and align with results from comparable research, reinforcing the validity of the research.

## Conclusion

Much of the literature on the use of escape rooms in nursing education is aimed at simulation within a clinical environment. Bringing these experiences into a university setting can open up a plethora of opportunities to engage nursing students and nurture professional values essential for clinical experiences.

This pilot study underscores the potential of escape rooms as an innovative and impactful teaching method in nursing education, promoting the key professional values; teamwork, decision-making, and information sharing. Through engaging students in dynamic, collaborative, and hands-on activities, escape rooms effectively bridge the gap between theoretical learning and practical application, catering to the evolving needs of nursing students, particularly those from Generation Z. The results show that escape rooms not only foster professional identity and teamwork but also offer an enjoyable and low stress learning experience, challenging traditional views on high-pressure educational environments.

As nursing education adapts to technological advancements and shifting generational dynamics, innovative strategies like escape rooms provide a modern, interactive, and inclusive approach to preparing students for the collaborative, fast-paced nature of clinical practice. While this study offers valuable insights, further research is needed to examine the scalability and adaptability of escape rooms in various educational contexts. Future investigations should focus on their long-term impact on clinical preparedness and the effectiveness across different generational and cultural groups. Additionally, longitudinal studies exploring their effect on professional development throughout nursing education and into clinical practice are essential. By refining and embracing such strategies, nursing education can continue to lead in preparing students for the evolving challenges of modern healthcare.

## Data Availability

The data that supports the findings of this study cannot be shared openly but is available upon reasonable request and with permissions from both authors.

## Declarations

### Accordance

No experiments were performed. The research was produced with ethical approval and informed consent from all participants was obtained.

### Minors

No minors participated in this study.
